# Supplementary material for: Left dorsolateral prefrontal cortex supports context-dependent prioritisation of off-task thought
Source: Nat Commun. 2019 Aug 23;10:3816. doi: 10.1038/s41467-019-11764-y (PMC6707151; doi:10.1038/s41467-019-11764-y)
Supplement: Supplementary file 1 — Supplementary Information [file 41467_2019_11764_MOESM1_ESM.pdf]

Dimensions	Questions	1	4
Task	My thoughts were focused on the task I was performing.	Not at all	Completely
Future	My thoughts involved future events.	Not at all	Completely
Past	My thoughts involved past events.	Not at all	Completely
Self	My thoughts involved myself.	Not at all	Completely
Person	My thoughts involved other people.	Not at all	Completely
Emotion	The content of my thoughts was:	Negative	Positive
Images	My thoughts were in the form of images.	Not at all	Completely
Words	My thoughts were in the form of words.	Not at all	Completely
Vivid	My thoughts were vivid as if I was there.	Not at all	Completely
Detailed	My thoughts were detailed and specific.	Not at all	Completely
Habit	This thought has recurrent themes similar to those I have had before.	Not at all	Completely
Evolving	My thoughts tended to evolve in a series of steps.	Not at all	Completely
Deliberate	My thoughts were:	Spontaneous	Deliberate

Supplementary Table 1. Mind wandering questions asked to each participant during MDES. The first question was always “Task” then the other 12 questions in a random order. The scores from these questions were entered into a PCA.

Thought component	Contrast	Cluster size	p-value	MNI coordinates
**Off-task**	**Main effect on-task**	**411**<br>**367**	**.0043**<br>**.0069**	**(34, -60, 44)**<br>**(-30, -70, 44)**
	**0-back off-task, 1-back on-task**	**198**	**.0308**	**(-26, 40, 30)**
Detail	Main effect negative detail	285<br>243	.0291<br>.0449	(-32, -48, 44)<br>(-8, -62, 54)
	Detail in 1-back, negative detail in 0-back	222	.0258	(-8, -50, 22)
Emotion	Main effect positive emotion	726<br>253	.0001<br>.0196	(40, 16, -16)<br>(-28, 24, -22)
**Task contrasts**	**Contrast**	**Cluster size**	**p-value**	**MNI coordinates**
	**0-back>1-back**	**7878**<br>**5397**<br>**4269**<br>**545**<br>**359**<br>**211**<br>**202**	**<.0001**<br>**<.0001**<br>**<.0001**<br>**.0004**<br>**.0039**<br>**.030**<br>**.034**	**(-8, -60, 10)**<br>**(52, -30, 14)**<br>**(-10, 64, 2)**<br>**(36, -90, 0)**<br>**(-14, 20, 2)**<br>**(4, -88, -16)**<br>**(-56, -30, 8)**
	**1-back>0-back**	**1160**<br>**880**<br>**504**<br>**186**	**<.0001**<br>**<.0001**<br>**.0007**<br>**0.0436**	**(-42, 2, 28)**<br>**(-28, -62, 42)**<br>**(-4, -2, 56)**

Supplementary Table 2. All significant clusters from the task-based analysis with multi-dimensional experience sampling. The primary analysis is shown in bold, the results from the other components are including from follow-up analyses.

Network 5 - 17 interaction					
Mask	Thought	Task	F-value	Equivalent r-value	Equivalence test p-value
DLPFC	Off-task	0	12.79	0.2942	Significant result: used to define effect size
		1	2.343	0.130612	0.018
	Detail	0	0.356	0.00263	0.001
		1	0.773	0.005693	0.003
	Modality	0	0.775	0.005708	0.003
		1	2.865	0.144403	0.03
	Emotion	0	0.551	0.004065	0.002

		1	0.281	0.045657	0.001
On-task regions (bilateral parietal)	Off-task	0	0.121	0.029925	0.001
		1	0.549	0.063641	0.002
Whole brain	Off-task	0	0.097	0.026796	<.001
		1	0	0	<.001
Network 10 -16 interaction					
Mask	Thought	Task	F-value	Equivalent r-value	Equivalence test p-value
DLPFC	**Detail**	**0**	**11.229**	**0.277111**	**0.412**
		1	14.014	0.306667	Significant result
	**Off-task**	**0**	**7.145**	**0.2242**	**0.185**
		**1**	**6.440**	**0.213382**	**0.151**
	Modality	0	0.563	0.097401	0.002
		1	1.293	0.097401	0.007
	Emotion	0	0.432	0.056478	0.002
		1	0.125	0.030415	0.001
On-task regions (bilateral parietal)	Detail	0	0.441	0.057062	0.002
		1	0.214	0.039783	0.001
Whole brain	Detail	0	0	0	<.001
		1	0.183	0.036793	0.001

Supplementary Table 3. Equivalence tests to check the specificity of the significant interactions identified within DLPFC. Effects that cannot be dismissed as null when looking for an effect of the size of our significant findings are in bold. The effect of the network 5 – network 17 interaction is specific to DLPFC and off-task thought in the 0-back. The effect of the network 10 – network 16 interaction is specific to DLPFC, but is similarly related to detail in both tasks, and may also relate to off-task thought as this test was not significantly equivalent to 0.

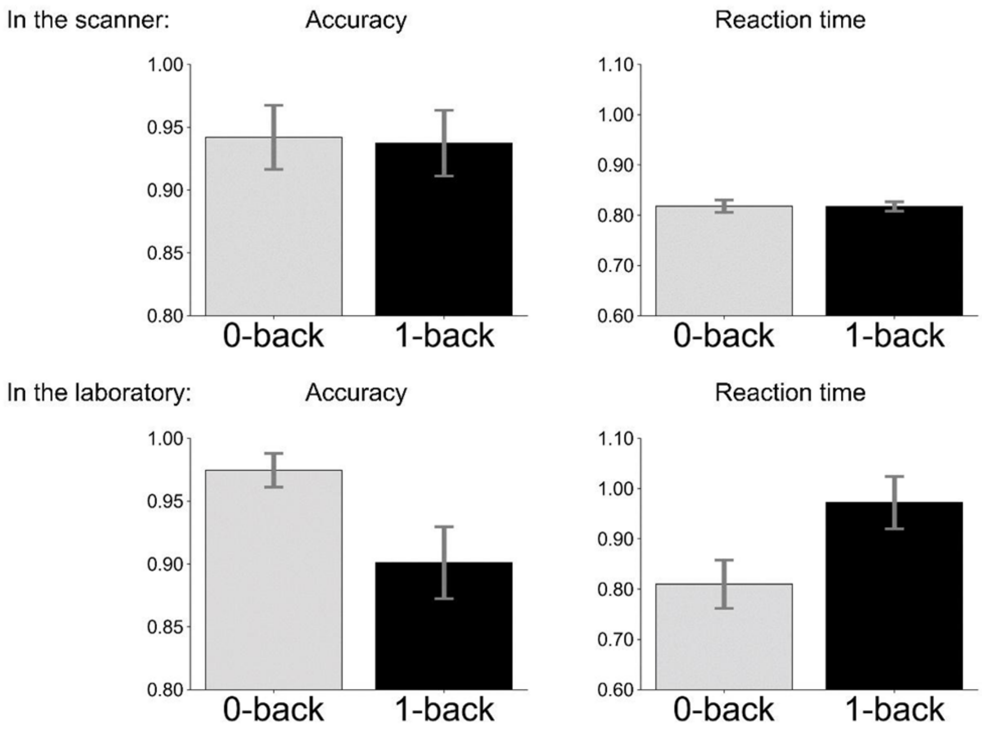

Supplementary Figure 1. Accuracy and reaction time in the scanner and laboratory during the 0- and 1-back tasks. Responses were significantly more accurate and faster during the 0-back in the laboratory, and there was no difference in the scanner.

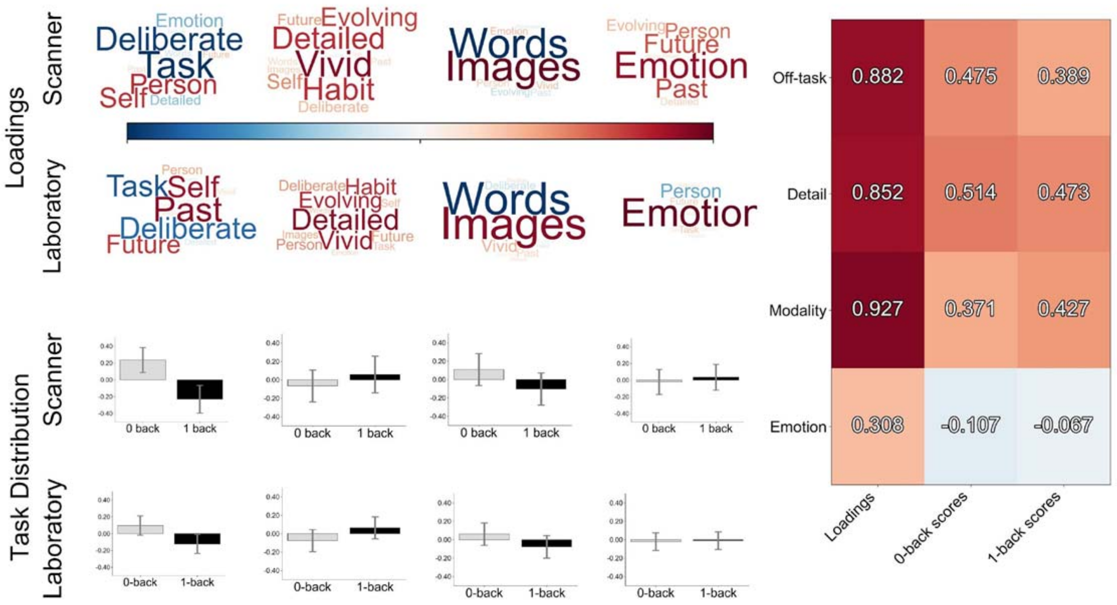

Supplementary Figure 2. PCA results characterised for scanner (n=60) and laboratory (n=146). Wordclouds show the loadings of each of the 4 components of thought (colourbar from -1 to 1). The components were labelled as (from left to right) Off-task, Detail, Modality, and Emotion. Bar charts show the levels of these thoughts across the two tasks. Heatmap represents the similarity in (from left to right) loadings (across 13 questions), scores in the 0 back, and scores in the 1 back (across individuals).

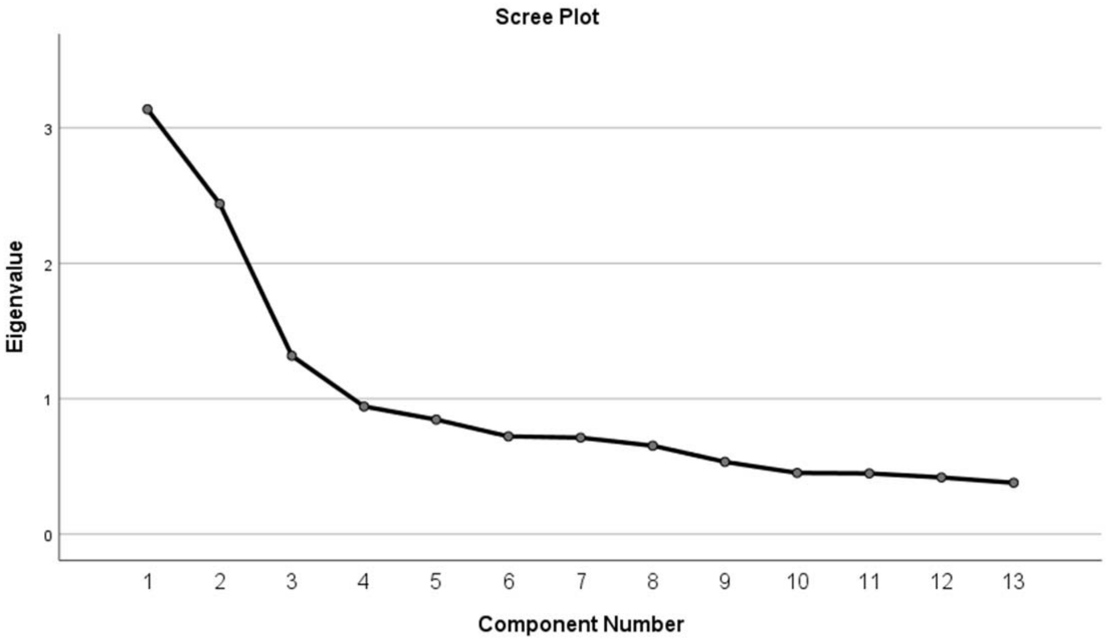

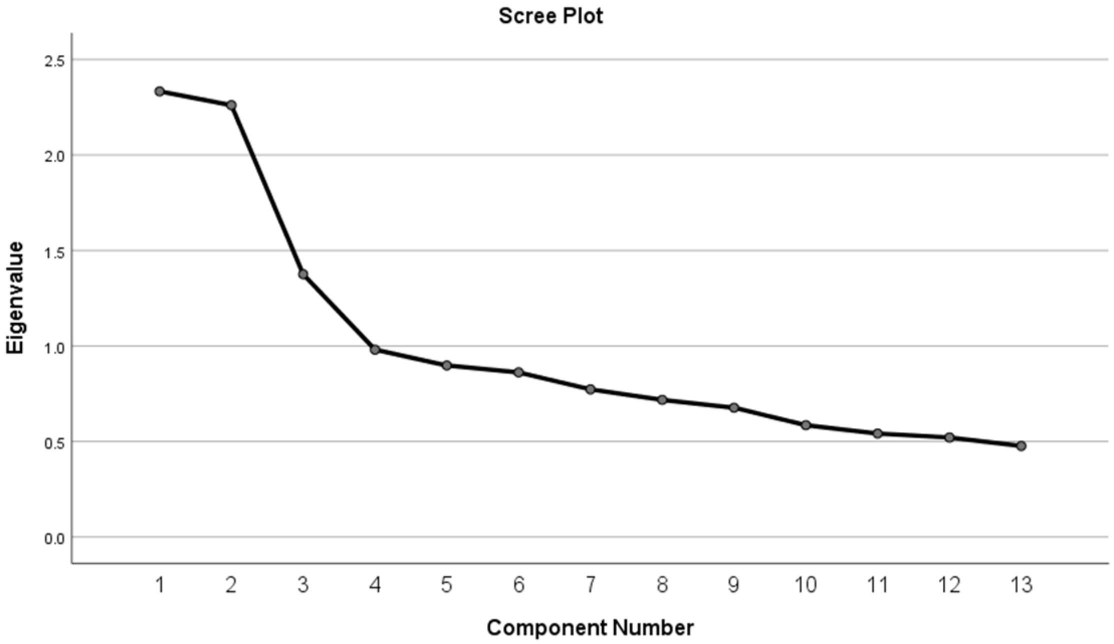

Supplementary Figure 3. Scree plots showing the eigenvalues of the 4 components of thought. The sharp change in slope following component 4 was used to select on 4 components for further analyses in both the scanner (top) and laboratory (bottom).

41

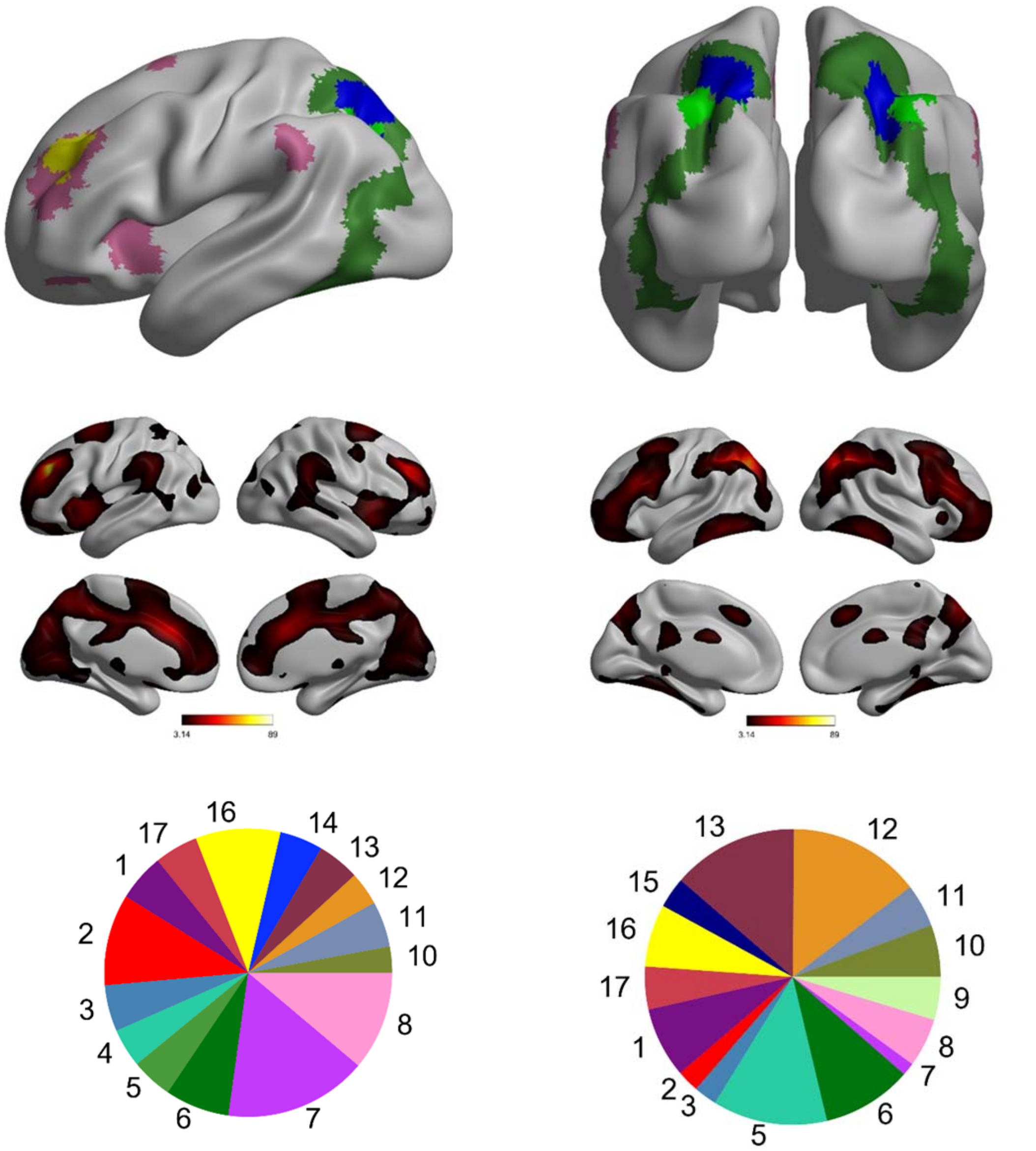

Supplementary Figure 4. Overlap of task-related regions with Yeo 17 networks, and pie charts showing the percentage of their resting state network that fell within each Yeo network[7]. The DLPFC region that was significantly more related to off-task thought in the 0 back and on-task thought in the 1-back fully overlaps (yellow) with Yeo 8 (pink), part of the Ventral Attention or Salience network. The bilateral parietal regions related to on-task thought in both tasks overlaps predominantly (blue, non-overlapping regions are shown in light green) with Yeo network 5 (dark green), which is part of the Dorsal Attention network. Similarly, the resting state connectivity of the DLPFC is predominantly in networks 7 and 8, making up the ventral attention network, while the superior parietal connectivity is largely in dorsal attention (5, 6) and frontoparietal control (12,13) networks.

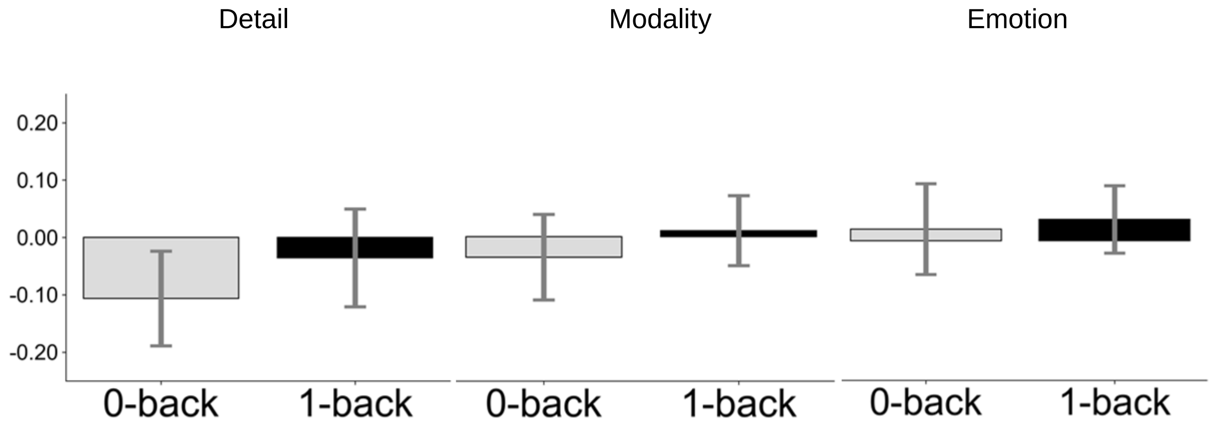

Supplementary Figure 5. Relationship of activity in the left DLPFC to the other components of thought. There was no task-dependent relationship (defined by the subtraction of the relationship in one task from the other) to any other component of thought, confirmed by equivalence analysis,

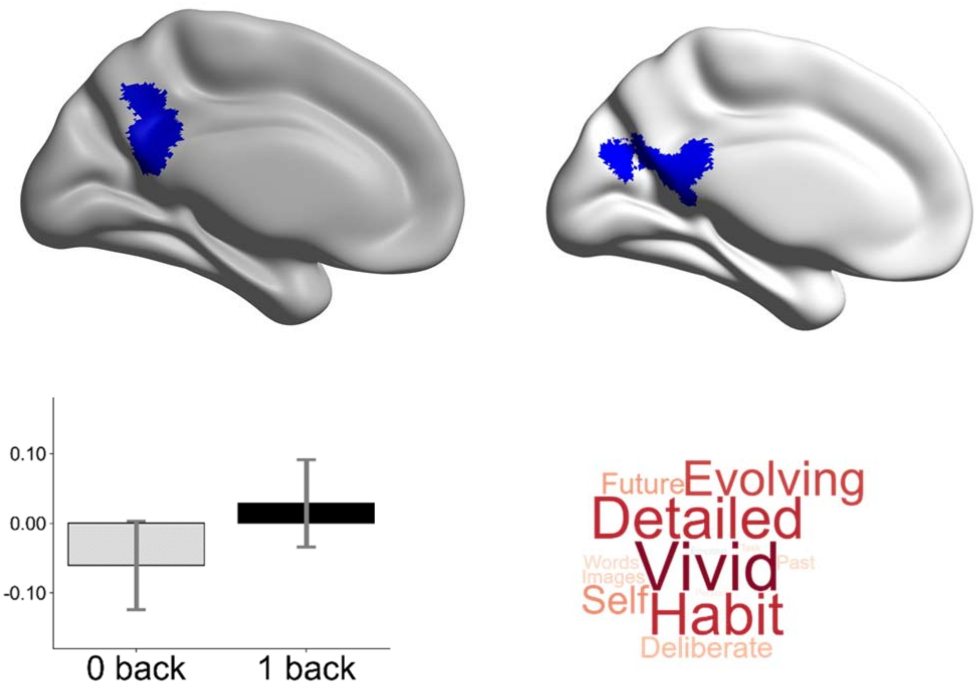

Supplementary Figure 6. Repeating the analysis with the other components of thought identified a region of the posterior cingulate cortex that was related to more detailed thought in the 1-back, and less detailed thought in the 0-back (top left). The association with detailed thought (wordcloud) in each task is shown in the bottom left. The brain on the top right shows an overlapping region of the brain that was identified as related to detailed thought in a previous study[23].

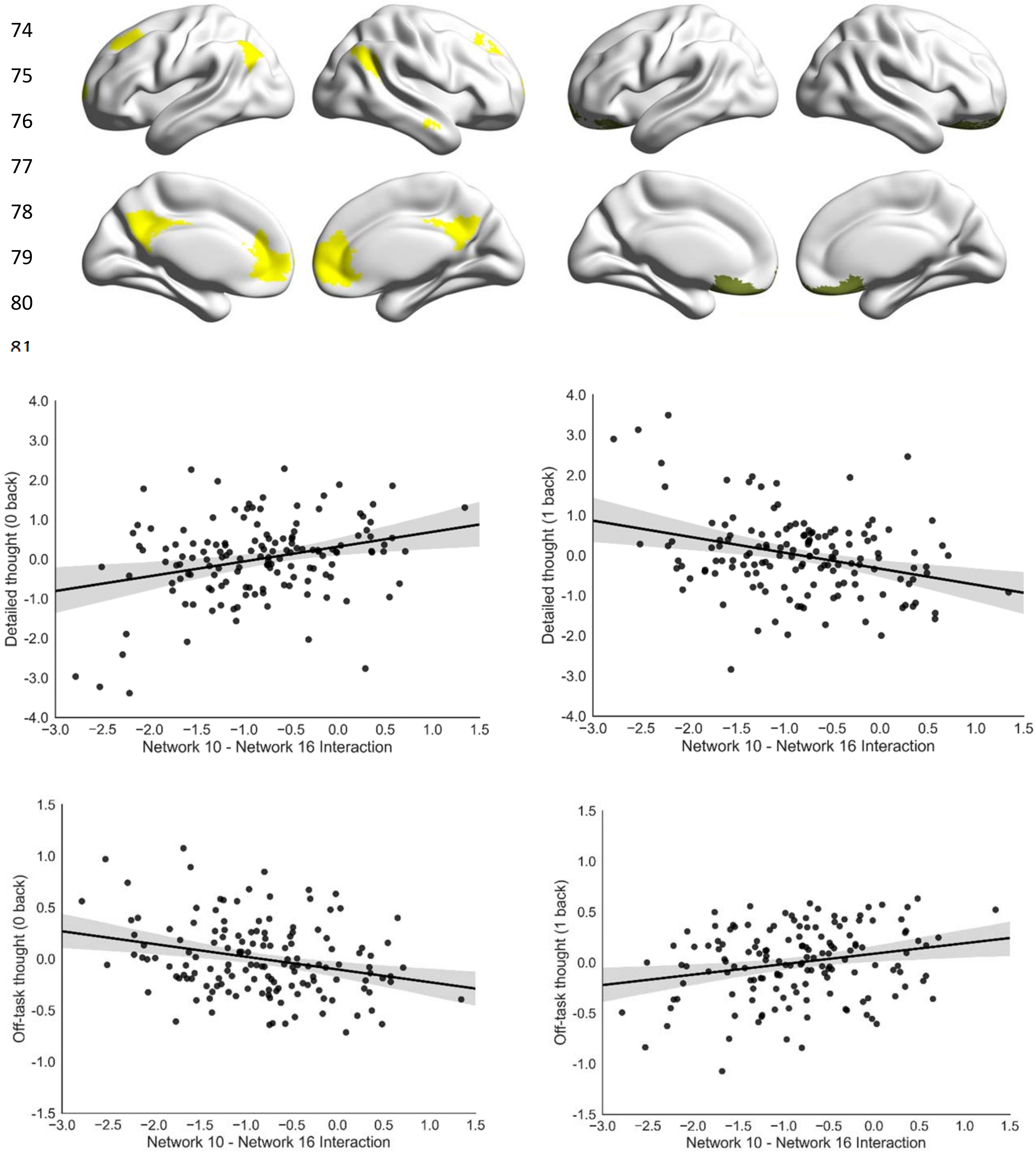

Supplementary Figure 7. The interaction between network 10 and network 16 within DLPFC is related to detailed thought in a task dependent manner. Stronger coupling between the network 10 and network 16 components within the DLPLC is related to more detailed thought in the 0-back, and less detailed thought in the 1-back. While not significantly passing Bonferroni correction, this interaction was also related to off-task thought with an effect size that could not be dismissed as null upon equivalence testing. This relationship is also task dependent, with the interaction negatively related to off-task thought in the 0-back, and positively in the 1-back. Network 10 is a region within the limbic system in vMPFC, and network 16 is the core of the DMN.

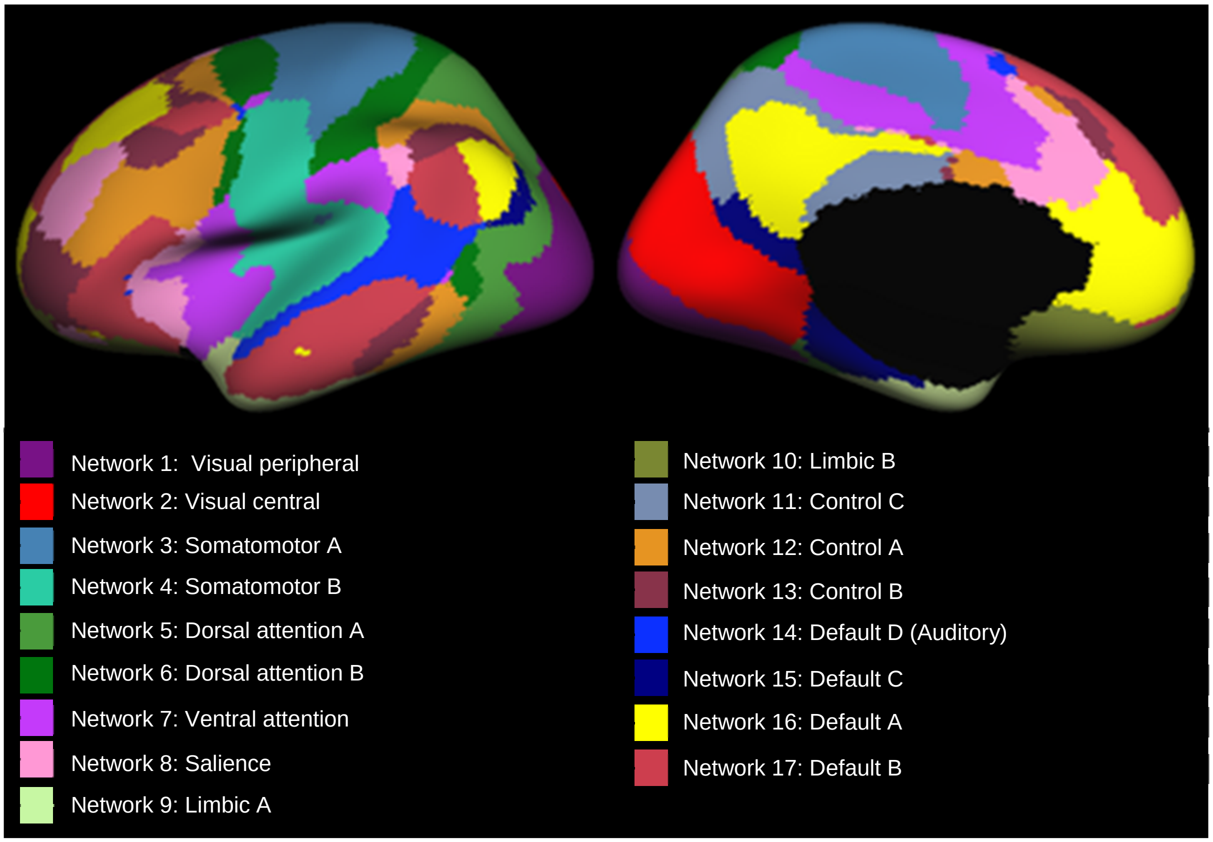

Supplementary Figure 8: Full description of the Yeo 17 networks. Names consistent with those used by: Baker, J. T., Holmes, A. J., Masters, G. A., Yeo, B. T., Krienen, F., Buckner, R. L., & Öngür, D. (2014). Disruption of cortical association networks in schizophrenia and psychotic bipolar disorder. JAMA psychiatry, 71(2), 109-118.

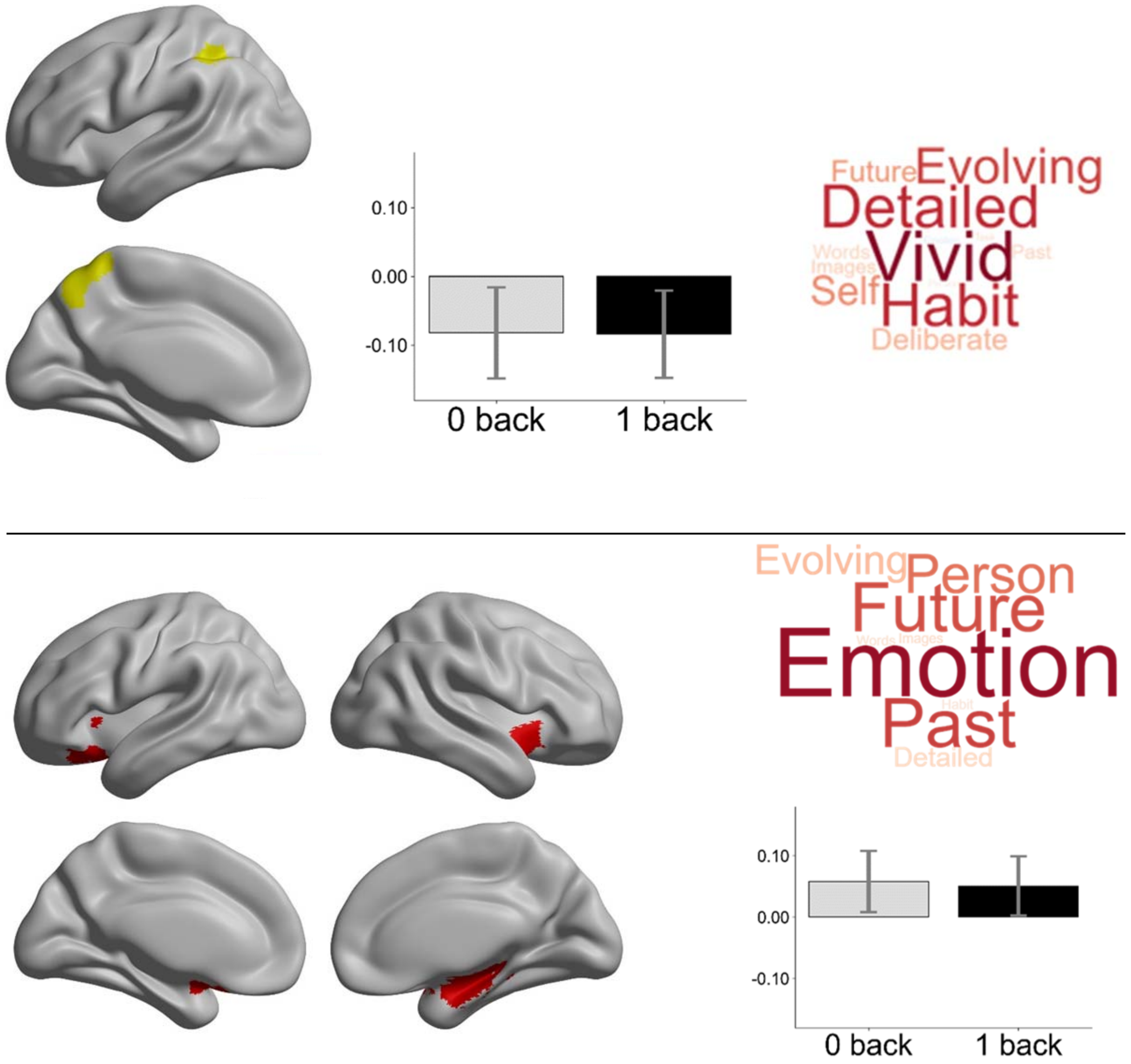

Supplementary Figure 9. No other results showed a pattern consistent with context regulation, but main effects identified brain regions related to vague thought (or negatively related to detail; top left) and positive emotional thought (bottom left).
