# Supplementary material for: Left dorsolateral prefrontal cortex supports context-dependent prioritisation of off-task thought
Source: Nat Commun. 2019 Aug 23;10:3816. doi: 10.1038/s41467-019-11764-y (PMC6707151; doi:10.1038/s41467-019-11764-y)
Supplement: Supplementary file 3 — Reporting Summary [file 41467_2019_11764_MOESM3_ESM.pdf]

nature research

Corresponding author(s): Adam Turnbull
Last updated by author(s): Jan 17, 2019

Reporting Summary

Nature Research wishes to improve the reproducibility of the work that we publish. This form provides structure for consistency and transparency in reporting. For further information on Nature Research policies, see Authors & Referees and the Editorial Policy Checklist .

Statistics

For all statistical analyses, confirm that the following items are present in the figure legend, table legend, main text, or Methods section.

n/a	Confirmed	
☐	☒	The exact sample size (n) for each experimental group/condition, given as a discrete number and unit of measurement
☐	☒	A statement on whether measurements were taken from distinct samples or whether the same sample was measured repeatedly
☐	☒	The statistical test(s) used AND whether they are one- or two-sided<br>*Only common tests should be described solely by name; describe more complex techniques in the Methods section.*
☐	☒	A description of all covariates tested
☐	☒	A description of any assumptions or corrections, such as tests of normality and adjustment for multiple comparisons
☐	☒	A full description of the statistical parameters including central tendency (e.g. means) or other basic estimates (e.g. regression coefficient) AND variation (e.g. standard deviation) or associated estimates of uncertainty (e.g. confidence intervals)
☐	☒	For null hypothesis testing, the test statistic (e.g. F, t, r) with confidence intervals, effect sizes, degrees of freedom and P value noted<br>*Give P values as exact values whenever suitable.*
☒	☐	For Bayesian analysis, information on the choice of priors and Markov chain Monte Carlo settings
☒	☐	For hierarchical and complex designs, identification of the appropriate level for tests and full reporting of outcomes
☒	☐	Estimates of effect sizes (e.g. Cohen's d, Pearson's r), indicating how they were calculated

Our web collection on statistics for biologists contains articles on many of the points above.

Software and code

Policy information about availability of computer code

Data collection	PyschoPy (version 1.85) was used to collect behavioural data. A 3T GE HDx Excite scanner was used to collect MRI data.
Data analysis	FSL (version 5), SPM (Version 12.0), MATLAB (version 16.a), CONN (version 17.f), SPSS (Version 24.0), FSL Nets (v0.6), FreeSurfer (version 5.3.0).

For manuscripts utilizing custom algorithms or software that are central to the research but not yet described in published literature, software must be made available to editors/reviewers. We strongly encourage code deposition in a community repository (e.g. GitHub). See the Nature Research guidelines for submitting code & software for further information.

Data

Policy information about availability of data

All manuscripts must include a data availability statement. This statement should provide the following information, where applicable:
- Accession codes, unique identifiers, or web links for publicly available datasets
- A list of figures that have associated raw data
- A description of any restrictions on data availability

Raw MRI data is confidential and restricted per our ethics agreement with the York Neuroimaging Centre. All summary data that support the findings in this paper are available on request. Unthresholded z-stat maps from the task-based fMRI portion of this study are available at https://neurovault.org/collections/3841/.

Field-specific reporting

Please select the one below that is the best fit for your research. If you are not sure, read the appropriate sections before making your selection.

Life sciences study design

All studies must disclose on these points even when the disclosure is negative.

Sample size	60 participants participated in the task-based portion of the study. 146 participants took part in the resting state part of this study. 39 of these participants completed both parts. Of the 146, 142 participants were included in the cortical thickness analysis. These sample sizes were chosen as relatively large samples for MRI research based on previous literature.
Data exclusions	Two participants were excluded for falling asleep, and one for excessive motion in the task-based portion of this study. Two participants were excluded for falling asleep and nine were excluded for excess motion in the resting state portion of this study. Four participants were excluded from the cortical thickness analysis after quality control of their surface projected data.
Replication	There were no direct replication attempts performed in this study, as the design and sample size are unique and relatively large. Several different imaging modalities were used to attempt to test the robustness of the relationships we identified between brain and behaviour.
Randomization	There were no between group analyses in this study.
Blinding	There were no between group analyses in this study.

Reporting for specific materials, systems and methods

We require information from authors about some types of materials, experimental systems and methods used in many studies. Here, indicate whether each material, system or method listed is relevant to your study. If you are not sure if a list item applies to your research, read the appropriate section before selecting a response.

Materials & experimental systems

n/a	Involved in the study	
☒	☐	Antibodies
☒	☐	Eukaryotic cell lines
☒	☐	Palaeontology
☒	☐	Animals and other organisms
☐	☒	Human research participants
☒	☐	Clinical data

Methods

n/a	Involved in the study	
☒	☐	ChIP-seq
☒	☐	Flow cytometry
☐	☒	MRI-based neuroimaging

Human research participants

Policy information about studies involving human research participants

Population characteristics	Task-based sample: 60 participants (37 females, mean age=20.17 years, S.D=2.22 years). Resting state sample: 146 participants (89 females, mean age=20.21 years, S.D=2.49 years). All participants were native English speakers, with normal/corrected vision, and no history of psychiatric or neurological illness.
Recruitment	Participants were recruited from the undergraduate and postgraduate population at the University of York.
Ethics oversight	Both experiments were approved by the local ethics committee at both the York Neuroimaging Centre and the University of York's Psychology Department.

Note that full information on the approval of the study protocol must also be provided in the manuscript.

Magnetic resonance imaging

Experimental design

Design type	This paper included a task-based study with a moderated block design, a resting state study, and a structural cortical thickness analysis.
Design specifications	Task-based: each run was 9-minutes in length and there were four runs per scanning session. In each run, there was an average of six thought probes (three in each condition), so that there were on average 24 (SD=3.30, mean=12 in each condition) MDES probes in each session.
Behavioral performance measures	Responses to the MDES probes were made using a button box with scores from 1 to 4 describing the levels of their thoughts. Responses were also made to target trials, but this data was not used for the analyses described in this paper.

Acquisition

Imaging type(s)	Functional and structural.
Field strength	3T
Sequence & imaging parameters	Structural and functional data were acquired using a 3T GE HDx Excite MRI scanner with an eight-channel phased array head coil tuned to 127.4 MHz. Structural MRI acquisition was based on a T1-weighted 3D fast spoiled gradient echo sequence (TR=7.8s, TE=minimum full, flip angle=20º, matrix size=256x256, 176 slices, voxel size =1.13x1.13x1mm). Functional data were recorded using single-shot 2D gradient echo planar imaging (TR=3s, TE=minimum full, flip angle=90º, matrix size=64x64, 60 slices, voxel size=3mm isotropic, 180 volumes). A FLAIR scan with the same orientation as the functional scans was collected to improve coregistration between scans.
Area of acquisition	Field of view covered the whole brain.

Diffusion MRI ☐ Used ☒ Not used

Preprocessing

Preprocessing software	FSL (version 5) was used to pre-process the task-based data. SPM (version 12.0) and CONN (version 17.f) was used on the resting state data. FreeSurfer (version 5.3.0) was used for structural data.
Normalization	Task-based: After coregistration to the structural images, individual functional images were linearly registered to the MNI-152 template using FMRIB's Linear Image Registration Tool (FLIRT). Resting state: Structural images were coregistered to the mean functional image via rigid-body transformation, segmented into grey/white matter and cerebrospinal fluid probability maps, and images were spatially normalized to the MNI-152 template.
Normalization template	MNI-152.
Noise and artifact removal	Task-based: The individual subject analysis first involved motion correction using MCFLIRT and slice-timing correction using Fourier space time-series phase-shifting. Functional images were spatial smoothed using a Gaussian kernel of FWHM 6mm, underwent grand-mean intensity normalisation of the entire 4D dataset by a single multiplicative factor, and both highpass temporal filtering (Gaussian-weighted least-squares straight line fitting, with sigma=100s); and Gaussian lowpass temporal filtering, with sigma=2.8s. Motion was additionally included as a group-level regressor. Resting state: The individual subject analysis first involved motion correction with six degrees of freedom and slice-timing correction. In additional to the removal of six realignment parameters and their second-order derivatives using a GLM, a linear detrendingterm was applied as well as the CompCor method with five principle components to remove signal from white matter and cerebrospinal fluid. Motion was included as a group level regressor.
Volume censoring	Volumes affected by motion were identified and scrubbed if motion exceeded 0.5mm or global signal changes were larger than z=3. Nine participants that had more than 15% of their data affected by motion were excluded from the analysis.

Statistical modeling & inference

Model type and settings	A model was set up for off-task thought by including four explanatory variables (EVs) as follows: EVs 1 and 2 modelled time periods in which participants completed the 0-back and 1-back task conditions; EVs 3 and 4 modelled the three thought probes in each condition, respectively, with a time period of 6 seconds prior to the MDES probe and the scores for the task-related component.
Effect(s) tested	Contrasts were included to assess brain activity that related to each task, as well as each component of thought. For the tasks, 0-back>1-back and 1-back>0-back contrasts were included. For the thoughts, main effects (positively or negatively related to thoughts in both conditions) and comparisons (activity related to thoughts in 0-back>thoughts in 1-back and vice versa) were included. The four runs were included into a fixed level analysis to average across the activity within an individual.

Specify type of analysis: ☐ Whole brain ☐ ROI-based ☒ Both

Anatomical location(s)	ROIs for the resting state and structural analysis were defined from results of the task-based experiment.
Statistic type for inference (See Eklund et al. 2016)	Group level analyses were carried out using a cluster-forming threshold of Z>3.1.
Correction	Whole-brain correction at p<.05 FWE-corrected.

Models & analysis

n/a	Involved in the study
☐	☒ Functional and/or effective connectivity
☒	☐ Graph analysis
☐	☒ Multivariate modeling or predictive analysis

Functional and/or effective connectivity	The first step of the analysis involved entering these networks into a dual regression that regressed the timeseries from within each Yeo network and subsequently regressed these against each subjects 4D dataset within the region-of-interest. Combining these results across the group gives a map within each region-of-interest showing how much each voxel relates to each Yeo 17 network (or an "echo" of each network within this region). These maps were again merged into a single file and entered into the first step of a second dual regression in order to extract the timeseries of each Yeo 17 "echo" or component. FSL Nets (v0.6) was used to extract these timeseries and produce a matrix of interactions defined by the partial correlation.
Multivariate modeling and predictive analysis	These interactions were entered into a model as dependent variables to model their relationship to the average component scores (e.g. task-relatedness) in each task from the laboratory. Any interactions involving networks that did not pass voxel-wise correction at p<.05/17 (i.e. were not "echoed" significantly within the region) were excluded from this analysis. The average scores from each task were included as independent variables in the model (MANOVA), as well as the interaction between the scores in each task, and age, gender, and mean motion in order to additionally control for the effect of these covariates of no interest.
